# More Than a Decade of Atherosclerotic Cardiovascular Disease Polypill Trials: Lessons for Heart Failure Polypills

**DOI:** 10.1016/j.jacadv.2024.101320

**Published:** 2024-10-23

**Authors:** Alice Huang, Adam Hively, Abdul Salam, Dorairaj Prabhakaran, H Asita de Silva, Anushka Patel, Anthony Rodgers, Mark D. Huffman, Anubha Agarwal

**Affiliations:** a Department of Medicine and Global Health Center, Washington University in St. Louis, St. Louis, United States; b The George Institute for Global Health, University of New South Wales, Hyderabad, India; c Prasanna School of Public Health, Manipal Academy of Higher Education, Manipal, India; d Centre for Chronic Disease Control, New Delhi, India; e Clinical Trials Unit, Department of Pharmacology, Faculty of Medicine, University of Kelaniya, Ragama, Sri Lanka; f The George Institute for Global Health, University of New South Wales, Sydney, Australia

Atherosclerotic cardiovascular disease (ASCVD) is the leading cause of mortality worldwide. The concept of polypills targeting multiple modifiable risk factors for prevention of cardiovascular disease was first introduced in 2000 by Wald and Law.^1^ More than 2 decades later, there have been multiple, large-scale, randomized clinical trials (RCTs) evaluating the efficacy and safety of polypills with at least 1 blood pressure-lowering drug and 1 lipid-lowering drug on clinical outcomes. Synthesis of evidence from these RCTs led to a decision by the World Health Organization to include polypills for high-risk primary and secondary prevention of cardiovascular disease in the 23rd Model List of Essential Medicines.^2^ Polypills for heart failure with reduced ejection fraction (HFrEF) containing guideline-directed medical therapy in 1 pill have been proposed, and there are few early-stage trials ongoing.^3^ As the concept of HFrEF polypills gains traction, we aim to understand lessons learned from ASCVD polypill trials to inform strategies for HFrEF polypill research.

**What is the clinical question being addressed?** What are the lessons learned from polypill trials for prevention of atherosclerotic cardiovascular disease to inform strategies for polypill research for heart failure with reduced ejection fraction?

**What is the main finding?** Key lessons learned include 1) global trial coordination, 2) incorporation of mixed methods research to inform implementation and scale-up, 3) understanding pathways to commercialization, 4) innovative patent pooling methods to enable equitable access while preserving commercial incentive, and 5) the need for large-scale global financing for cardiovascular disease to catalyze innovation.

We conducted a secondary analysis of a systematic review by including 10 RCTs of ASCVD polypills with at least 12 months of participant follow-up, and additional details of the systematic review methods are reported elsewhere.^1^ We extracted relevant data from the published manuscripts for each RCT related to trial design, funding, polypill manufacturing, regulatory and implementation challenges noted in published manuscripts, and impact metrics. Data are summarized using a narrative qualitative approach. No institutional review board approval was needed as this secondary analysis is not considered human subjects’ research.

Summary of characteristics of included RCTs are reported in Table 1. Most trials had diverse funding sources including pharmaceutical companies, universities, foundations, and governmental funding agencies. The time from participant recruitment to publication of primary results ranged from 3 years to >10 years for trials with longer follow-up periods, not including pretrial planning time. Polypills used in some trials were compounded with active pharmaceutical ingredients, and others were manufactured by overencapsulation of individual tablets. Some large-scale international trials, such as TIPS-3, faced regulatory challenges in different countries, including varied approval times and implementation challenges related to restrictions on polypill importation and drug resupply resulting in delays.^4^ Many trials included process evaluation studies using mixed methods to understand patient and provider opinions of the polypill within the trial context. The suite of trials led to a synthesis of evidence that supports adoption and implementation of polypills to lower risk of ASCVD events (relative risk 0.71, 95% CI 0.63-0.79) and all-cause mortality (relative risk 0.89, 95% CI 0.78-1.00).^1^

**Table 1:** Characteristics of Included Trials

Study Characteristics	Funding	Timeline From Participant Recruitment to Publication	Polypill Manufacturing	Implementation and Regulatory Challenges	Citations and Secondary Analyses
Malekzadeh (2010) 2-group RCT N = 475 Mostly primary prevention	Tehran University of Medical Sciences, Iranian Ministry of Health and Medical, and the Alborz Daru Pharmaceutical Company	4.5 y (January 2006-August 2010)	Combination polypills manufactured by Alborz Daru Pharmaceutical Company (Iran)	Participant drop-out: 26.7% (127 out of 475 participants did not complete the 12 mo of follow-up).	Citations^a^ = 186 Smaller trial that preceded a large outcomes trial, PolyIran 2019
CRUCIAL (2011) 2-group cluster RCT N = 1,461 Mostly primary prevention	Pfizer Inc	4 y (March 2007-February 2011)	Single-pill combination polypills manufactured by Viatris Inc (United States)	Participant drop-out: 9% 4 investigators withdrew from the study before initiating treatment for any of their patients.	Citations = 69
UMPIRE (2013) 2-group RCT N = 2004 Mixed secondary prevention	European Commission Seventh Framework Programme	3 y (July 2010-September 2013)	Combination polypills manufactured by Dr Reddy’s Laboratories (India)	Maintaining consistent polypill storage conditions across international sites.	Citations = 481 Process evaluation, SPACE collaboration individual patient data meta-analysis
Kanyini GAP (2014) 2-group RCT N = 623 Mixed secondary prevention	National Health and Medical Research Council of Australia	5.5 y (January 2010-July 2015)	Combination polypills manufactured by Dr Reddy’s Laboratories (India)	Participant recruitment due to insufficient resources.	Citations = 174 Process evaluation, SPACE collaboration individual patient data meta-analysis, cost-analysis
IMPACT (2014) 2-group RCT N = 513 Mixed secondary prevention	New Zealand Health Research Council, National Heart Foundation of New Zealand, New Zealand Lotteries Grants Board, University of Auckland, New Zealand’s Pharmaceutical Management Agency, Auckland regional district health boards, Auckland Medical Research Foundation	4 y (July 2010-May 2014)	Combination polypills manufactured by Dr Reddy’s Laboratories (India)	Polypill discontinuation: 37% discontinued the polypill treatment in the intervention arm, primarily due to side effects.	Citations = 215 Process evaluation, SPACE collaboration individual patient data meta-analysis
Muñoz (2019) 2-group RCT N = 303 Mostly primary prevention	American Heart Association Strategically Focused Prevention Research Network, National Institutes of Health	4 y (December 2015-September 2019)	Overencapsulated polypills manufactured by the Vanderbilt University Investigational Drug Service (United States)	From 1,201 patients assessed for eligibility, only 303 were randomized. A significant number were excluded due to not meeting inclusion criteria, declining participation, or not responding to questionnaires.	Citations = 162
PolyIran (2019) 2-group cluster RCT N = 6,838 Mostly primary prevention	Tehran University of Medical Sciences, the Barakat Foundation, and Alborz Darou Pharmaceutical Company	8.5 y (February 2011-August 2019)	Combination polypills manufactured by Alborz Daru Pharmaceutical Company (Iran)	Clusters excluded due to challenges accessing rural villages.	Citations = 265 Individual patient data meta-analysis including cost-effectiveness analysis with HOPE-3 and TIPS-3
TIPS-3 (2021) 2-by-2-by2 factorial design RCT N = 5,713 Mostly primary prevention	Wellcome Trust, the Canadian Institutes for Health Research, Cadila Pharmaceuticals, Population Health Research Institute, Heart and Stroke Foundation of Ontario, Philippines Council for Health Research and Development, Secretaria de Salud del Departamento de Santander, and St. John's Research Institute	8.5 y (June 2012-January 2021)	Compounded polypills manufactured by Cadila Pharma (India)	Regulatory approval times in different countries ranged from 3 mo to 1 y, and ethics submissions were withdrawn from select countries after facing multiple challenges for study approval. Unanticipated regulatory challenges, restrictions on drug importation, and drug resupply resulted in significant delays.	Citations = 240 Economic cost-effectiveness evaluation, individual patient data meta-analysis with HOPE-3 and PolyIran
SECURE (2022) 2-group RCT N = 2,499 Exclusive secondary prevention	European Union Horizon 2020	7 y (August 2016-August 2022)	Combination polypills manufactured by Ferrer International (Spain)	All patients were enrolled by the end of 2019 before the start of the COVID-19 pandemic. However, the pandemic may have precluded some patients from completing trial visits due to site closures, travel restrictions, and stay-at-home requirements during 2020.	Citations = 176 Cost-effectiveness evaluation planned
PolyIran-Liver (2022) 2-group pragmatic RCT N = 2,266 Mostly primary prevention	Tehran University of Medical Sciences, the Barakat Foundation, and Alborz Darou Pharmaceutical Company	10 y (October 2011-December 2021)	Combination polypills manufactured by Alborz Daru Pharmaceutical Company (Iran)	Less participation in the control group as compared to the intervention group in the final follow-up visit (56% vs 76%) when laboratory/imaging studies were repeated.	Citations = 16

RCT = randomized clinical trial.

a Citations as of June 2024 by PubMed, National Library of Medicine, National Institutes of Health.

A key lesson for polypill research for HFrEF is the need for greater global coordination with public-private partnerships to conduct large-scale, efficient RCTs to answer research questions for not only regulatory approval but also for implementation to maximize return on investment given the significant regulatory and operational hurdles specific to polypill trials. Type I or type II hybrid-effectiveness trial designs incorporating mixed methods to understand contextual factors that influence implementation can inform scale-up and sustainment of the polypill intervention beyond the trial funding period. Another essential lesson from ASCVD polypill trials is the importance of understanding the pathway to commercialization by partnering with industry like the Centro Nacional de Investigaciones Cardiovasculares and Ferrer public-private partnership to develop Trinomia/Sincronium, a polypill including aspirin, ramipril, and atorvastatin for secondary prevention of ASCVD.^5^ These public-private partnerships also support trial implementation, suggesting they should be preferentially explored in HFrEF polypill RCTs.

Polypills that create new intellectual property that can be protected by patents, such as long-acting injectable combinations for cardiovascular disease, may be more likely to be commercially viable. Innovative approaches to patent pooling and licensing, such as the Medicines Patent Pool, can enable equitable access to new polypills while preserving commercial incentives for investment by pharmaceutical companies. Lastly, alternative financing strategies such as a “Global Fund” for cardiovascular disease, modeled after the Global Funds for AIDS, tuberculosis, and malaria prevention and treatment, could align interests at multiple levels to catalyze future polypill innovation. These lessons learned from more than a decade of ASCVD polypill trials provide a scaffold to inform future research on HFrEF polypills to help realize their potential in improving patient outcomes and population health.

## Funding support and author disclosures

Dr Agarwal is supported by 10.13039/100000050NIH/NHLBI Grant K99HL157687. Dr Huffman has received travel support from the 10.13039/100000968American Heart Association and 10.13039/501100015708World Heart Federation and consulting fees from PwC Switzerland. Drs Huffman, Salam, Patel, and Rodgers have an appointment at The George Institute for Global Health, which has a patent and license and has received investment funding with intent to commercialize fixed-dose combination therapy through its social enterprise business, George Medicines. Drs Huffman and Agarwal have pending patents for heart failure polypills. All other authors have reported that they have no relationships relevant to the contents of this paper to disclose.

## References

[1] Agarwal A., Mehta P.M., Jacobson T. (2024). Fixed-dose combination therapy for the prevention of atherosclerotic cardiovascular disease. Nat Med.

[2] World Health Organization (2023). WHO EML 23rd list. https://iris.who.int/bitstream/handle/10665/371090/WHO-MHP-HPS-EML-2023.02-eng.pdf?sequence=1.

[3] Agarwal A., Yancy C.W., Huffman M.D. (2020). Improving care for heart failure with reduced ejection fraction—a potential polypill-based strategy. JAMA.

[4] Yusuf S., Joseph P., Dans A. (2021). Polypill with or without aspirin in persons without cardiovascular disease. N Engl J Med.

[5] Webster R., Castellano J.M., Onuma O.K. (2017). Putting polypills into practice: challenges and lessons learned. Lancet.

